# Exploring factors affecting the adoption and use of digital health technologies among older adults with cancer: A qualitative study

**DOI:** 10.1007/s00520-025-09813-y

**Published:** 2025-08-06

**Authors:** Misun Hwang, Youmin Cho, Katie Gahn, Heidi Mason, Milisa Manojlovich, Yang Gong, Yun Jiang

**Affiliations:** 1 University of Michigan School of Nursing, 400 North Ingalls Building, Ann Arbor, MI USA; 2 Chungnam National University College of Nursing, Daejeon, South Korea; 3 McWilliams School of Biomedical Informatics, University of Texas Health Science Center, Houston, TX USA

**Keywords:** Cancer, Oncology, Older adults, Digital health, Qualitative study

## Abstract

**Purpose:**

Although digital health technologies (DHTs) are promising to improve health outcomes in older adults with cancer, the low adoption and limited use remain significant gaps in their effective digital health care. Little is known about their concerns about adopting and using DHTs in routine life, particularly in the continued use phase. This study aims to explore factors affecting the initial adoption and continued use of DHTs among older adults with cancer.

**Methods:**

A secondary analysis of qualitative data was conducted based on interviews with 21 older adults (≥ 65 years) with breast, prostate, lung, or colorectal cancer. The transcripts of interview recordings were analyzed using a thematic analysis.

**Results:**

Three major themes and several subthemes were identified as potential factors affecting the (1) initial adoption, (2) continued use, and (3) limited use of DHTs. Digitalized healthcare systems and access to technology influenced the initial adoption of DHTs. Perceived ease of use, perceived usefulness, expected timely care from providers, and increased sense of control emerged as leading factors to the continued use. The limited use of DHTs was influenced by a lack of knowledge and skills, a lack of direct interaction with providers, and concerns about digital communication quality.

**Conclusions:**

Ensuring digital access and providing technology-based solutions that meet diverse patient needs is crucial to promoting the adoption and use of DHTs among older adults with cancer. Healthcare providers should address older adults’ low digital literacy and uncertainty to ensure the quality of cancer care provided through DHTs.

## Introduction

With the aging of the population, older adults (≥ 65 years old) are projected to represent approximately 60% of the global cancer incidence by 2035 [1]. Older adults with cancer often have complex and multifaceted healthcare needs due to comorbidities, reduced functional status, and other aging-related conditions [2–4]. This complicates cancer management for this population and increases the risk of treatment-related toxicities, unplanned hospitalization, and mortality [5, 6]. Additionally, since cancer is primarily managed in outpatient clinical settings, older adults with cancer are expected to take on the responsibility of managing their treatment and underlying diseases, maintaining self-management behaviors, and coping with the psychosocial impact of their illness at home [7, 8]. Given the rapidly growing population of older adults with cancer and their unique care demands, it is crucial to provide support that helps older adults actively monitor and manage their health and health care throughout their cancer journey [9].

The application of digital health technologies (DHTs), such as mobile applications, web-based platforms, and wearable devices, holds significant potential to improve health outcomes for older adults with cancer [10]. DHT-based interventions have been associated with improved self-management behaviors (e.g., physical activity, treatment adherence, diet), symptom management, and quality of life in older adults with cancer by supporting the early detection of symptoms, monitoring and managing adverse effects, and promoting healthy behaviors [6, 11, 12]. A body of literature has demonstrated that DHTs can empower older adults to be more engaged with their cancer treatment and enhance patient-provider communication [13, 14].

Despite the availability and potential benefits of DHTs, there remain significant gaps in their effective integration into routine cancer care due to low adoption and use by older adults. Data from the 2022 National Health and Aging Trend Study showed that nearly half of older adults with cancer reported non-use of any DHTs in the United States [15]. Prior studies have demonstrated that older adults with cancer do not utilize DHTs to an optimal level even after their adoption [13, 16, 17]. Given that the effectiveness of DHTs relies on user engagement [18], promoting their adoption and use is essential to ensure older adults with cancer fully benefit from them. Additionally, older adults with cancer represent unique preferences and high variability in utilizing health information and technology, which emphasizes the need for tailored solutions based on a comprehensive understanding of their perceptions of DHTs [19, 20].

While existing studies have explored perceptions of technology use in this population, most have focused on the adoption phase, assessing patients’ views of the usability or acceptability of specific technologies [19, 21] or have broadly explored perceptions without differentiating factors affecting the adoption and usage phase [22, 23]. Little is known about how older adults with cancer adopt and use DHTs in their daily care, particularly in the post-adoption phase. Understanding patients’ perceptions of DHTs, considering their ongoing experience from adoption to post-adoption use, can help identify and differentiate influencing factors at each phase and provide tailored support. Therefore, this study aims to explore factors affecting the initial adoption and continued use of DHTs among older adults with cancer.

## Methods

### Design and procedure

This is a secondary analysis study of interview data from a larger qualitative study to explore factors influencing the willingness of patients with cancer to report medication safety concerns from home [24]. The parent study conducted in-depth qualitative interviews (N = 41) with patients who received care at the University of Michigan Rogel Cancer Center [24]. This secondary analysis analyzed interview transcripts from participants aged ≥ 65 at the time of the interview (N = 21), focusing on their unique experiences and perceptions of using digital health technologies.

### Sample

Participants were recruited via the University of Michigan Rogel Cancer Center. The inclusion criteria were: (1) age ≥ 18 years old; (2) diagnosis of breast, colorectal, lung, and prostate cancers; and (3) experience of care transition from hospitals to home in the past year. The original data were collected between April and December 2022, and individual semi-structured phone interviews were conducted by trained research staff. Informed consent was obtained from all individual participants included in the study. The interview guide covered topics on daily life, medication management, side effects, the impact of COVID-19, and technology use. Details regarding the recruitment and data collection process have been published elsewhere [24].

### Data analysis

The transcripts were organized in Word files, and qualitative data management software NVivo 12 (QRS International, Melbourne, Australia) was used. Transcripts were analyzed by the first author (MH) and an experienced qualitative researcher (KG) using a thematic analysis approach [25]. The two researchers examined the initial coding data, and discrepancies between the coders were discussed until a mutual agreement was reached. Similar codes were grouped into subthemes based on their relationships, and the subthemes were classified into themes. Comparable interpretations of the themes and subthemes were discussed during weekly research team meetings, which involved an oncology nurse practitioner, qualitative researchers, and digital health technology experts to reduce potential biases in the representation of findings.

## Results

A total of 21 interview transcripts were analyzed in this study. The characteristics of study participants are presented in Table 1.

**Table 1:** Characteristics of study participants

**Characteristics**	**Sample**
Age (by years), mean (SD)	71.7 (3.7)
**Age group, *n* (%)**	
65–74	15 (71.4)
75–84	6 (28.6)
**Cancer type, *n* (%)**	
Prostate	9 (42.9)
Colorectal	5 (23.8)
Breast	4 (19.0)
Lung	3 (14.3)
**Gender, *n* (%)**	
Male	11 (52.4)
Female	10 (47.6)
**Race**	
White	18 (85.7)
Black or African American	3 (14.3)
**Marital status**	
Married	16 (76.2)
Unmarried, divorced, or widowed	5 (23.8)
**Educational background**	
High school or others	2 (9.5)
Some college	9 (42.9)
College graduate	3 (14.3)
Postgraduate	7 (33.3)
**Occupational status**	
Full-time or part-time	4 (19.0)
Retired	17 (81.0)

SD, standard deviation

Three major themes emerged from the exploration of patient perceptions of DHTs among older adults with cancer: (1) Factors affecting the initial adoption of DHTs, (2) Factors affecting the continued use of DHTs, and (3) Factors affecting the limited use of DHTs. Each theme included several subthemes, as below.

### Factors affecting the initial adoption of digital health technologies

Participants shared factors that impacted their decision to initially adopt DHTs for cancer management. While the digitalized healthcare system, driven by the COVID-19 pandemic, facilitated the adoption of DHTs in general, a lack of access to technology was a significant barrier for some participants.

#### Digitalized healthcare system

Participants described adopting DHTs because the traditional healthcare system became digitalized, partially because of the COVID-19 pandemic. They began virtual visits to meet their providers as cancer care delivery changed, regardless of their preferences.

*“I started doing it when COVID started because I was scheduled for some appointments at the very beginning of the pandemic. They were even just delayed, so they moved quickly to having one or two of the appointments by Zoom.” (Patient 14)*

Participants mentioned that healthcare providers’ attitudes toward using technology had also changed within the digitalized healthcare system.

*“I never did video visits or telephone visits before the pandemic. They also weren't as readily available. A lot of doctors did not want to do video visits; they wanted it in person, and I understand that, and it was only because of the pandemic that they became widely available.” (Patient 2)*

#### Access to technology

Some participants emphasized the challenges they faced in adopting DHTs, citing barriers such as their geographic location or the financial burden of securing an internet connection and technological devices.

*“My phone wouldn't work just right. I live out in the country, and my computer wouldn't pick it up at all.” (Patient 1)*

*“I am living in a senior apartment building. Only about half of the people even have a computer, and only about two-thirds of those who have a computer have internet because they just simply can't afford it, so they're never going to have a video visit.” (Patient 2)*

### Factors affecting the continued use of digital health technologies

Participants shared several factors that affected their continued use of DHTs after initial adoption, including perceived ease of use, usefulness in saving time and cost, receiving timely care from healthcare providers, and increased sense of control. These perceptions helped participants continue using DHTs routinely for cancer management in their daily lives.

#### Ease of using technology

Participants expressed ease in using general or specific types of DHTs, such as patient portals or video conferencing. They felt that the more they used the technology, the more familiar and comfortable they became.

*“I’ve had a couple of video calls. I was comfortable with those, so I’m fine using that stuff.” (Patient 4)*

*“I’ve used that portal for so long for so many things that I'm used to using it.” (Patient 14)*

#### Usefulness in saving time and cost

Participants described the advantages of digital health technology in saving time and the cost associated with hospital visits. Participants saved time by not having to wait or drive, and they emphasized the cost-effectiveness of virtual visits compared to in-person hospital visits as a reason for using technology.

*“I see how virtual visits would be more beneficial, you don’t have the stress of driving to the doctor’s office and trying to find a parking space. It’s kind of a hassle.” (Patient 15)*

*“Generally, a visit to the hospital costs me five hundred dollars roughly, so I’d much rather have a video visit.” (Patient 2)*

#### Expected timely care from healthcare providers

Participants believed that using digital health technologies would help them address their health-related concerns promptly and in a timely manner through healthcare providers.

*“When I have a concern, and I make an inquiry, I receive a response in a timely way, and the response makes sense to me, and I've always had that experience on the portal.” (Patient 14)*

Participants underscored flexibility in terms of time. Using technology and receiving care whenever needed was satisfactory and perceived as a benefit.

*“The pros are you can use it at any time. I usually get a good response- a quick response, especially with my oncology office. I can’t think of any cons.” (Patient 6)*

#### Increased sense of control

Participants felt an increased sense of control regarding their health status through accessing and tracking test results, medical appointment schedules, and medical notes through digital health technologies. Participants stated that technology helps them to independently understand their status and engage in the treatment process.

*“I get test results on the portal, and I can often see my blood work or what the doctor says. I do a lot of independent research on my own. I'll look for stuff that isn't obvious, that hasn't been searched before, and that stuff just to gain knowledge and information.” (Patient 19)*

*“I like to know how my results look, not only good, but also how they went, what was being used, and what's going to be used in the future, if it's going to be used. So, I think the portal basically, for people like me who can use it and use it with interest, it's a fantastic solution.” (Patient 5)*

Participants described that technology provides additional opportunities for expressing their care needs and concerns in their own words.

*“In many cases, you can discuss it with them and get the information you need, and they get the information they want from you. I like the technology because it gives me a chance to try to explain as much as possible in my words.” (Patient 16)*

### Factors affecting the limited use of digital health technologies

Participants mentioned several factors that hindered them from using technology actively after initial adoption, including a lack of knowledge and skills, a lack of direct interaction with healthcare providers, and concerns about communication effectiveness and reliability. These perceptions influenced the limited use of DHTs in daily life.

#### Lack of knowledge and skills

Some participants mentioned their lack of knowledge and skills related to technology, mainly associated with their generational characteristics. They were introduced to technology at a relatively late age and remained unfamiliar, contributing to their limited use of DHTs.

*“I’m not a tech person, I’m 75 years old, and I could turn the computer on, I think it's more of my lack of knowledge and how to use the portal.” (Patient 20)*

Participants expressed challenges in using technology independently after initial adoption. Due to their limited knowledge and skills, they needed additional support from family caregivers, including spouses or children, in addressing technical issues or setting up devices.

*“I have two grown daughters who are, and they walk me through stuff or whatever, set up my video call” (Patient 4)*

#### Lack of direct interaction with healthcare providers

Participants expressed a need for direct interaction with healthcare providers through in-person visits, particularly if they experience changes in health status. While they were relatively comfortable using technology when their health status was stable, any changes, such as alterations in treatment plans or the occurrence of new symptoms, increased their desire for in-person visits.

*“If there was a change in my treatment or a change in the cancer, and we were going to be talking about serious options, I think I might want to be there in person.” (Patient 14)*

Some participants were concerned about the accuracy of solely relying on technology for self-reporting their health status. They believed healthcare providers needed to physically see their condition to ensure accurate assessments and treatments.

*“Because I’m no doctor. So, how can I say, “Oh, I’ve got this.” In my opinion, they need sometimes to see that as well. I didn’t like how they relied on the patient to tell them instead of seeing them.” (Patient 3)*

#### Concerns about digital communication quality

Participants expressed concern about the quality of communication via technology. Participants were uncertain whether healthcare providers recognized their health information or issues appropriately. Participants sometimes had negative experiences due to miscommunication with healthcare providers, contributing to a perception of ineffectiveness and nonadherence to technology use.

*“The thing I didn’t like or question was how they relied on the patient to tell them instead of seeing them. And what if my thermometer is not accurate? So, if I’m sick, I probably would want to go in now with my hospital experience.” (Patient 3)*

Some participants stated that digital communication lacked consistency, leading to uncertainty about whether information was delivered correctly to healthcare providers.

*“I’m not sure that things are getting handled. The information is getting passed on, but I’m not sure if it’s just a text message or if they actually make it a priority message.” (Patient 2)*

## Discussion

This qualitative study explored the factors affecting the adoption and use of DHTs from the perspectives of older adults with cancer. The identified themes were categorized into factors affecting the initial adoption, continued use, and limited use of DHTs. The findings provide insight into the unique preferences and challenges in both the adoption and post-adoption phases of DHT use among older adults with cancer.

Study findings indicate that healthcare system change and technology accessibility have influenced the decision of older adults with cancer to adopt DHTs. The COVID-19 pandemic forced oncology practices to limit face-to-face visits and increase virtual interactions [26], making technology use essential for maintaining cancer care in a digitalized healthcare system. This shift was further supported by policy and regulatory changes regarding telehealth services, such as the relaxation of patient eligibility and the preservation of reimbursement policies [27]. As a result, the prevalence of DHT use among older cancer survivors in the United States increased from 45% in 2019 to 52% in 2021 [15]. This trend was represented in our study participants, many of whom reported that they had never used technology before the pandemic but found adopting DHTs inevitable regardless of their preference. Meanwhile, several participants identified technology accessibility as a primary barrier to the initial adoption of DHTs, consistent with previous studies [28, 29]. Our study provides contextual evidence that the initial adoption of DHTs among older adults with cancer was driven by systemic support and healthcare providers’ attitudes rather than personal factors. This also highlights the need to improve digital access through better infrastructure and distribution, as accessibility remains a barrier at the individual level.

In the post-adoption phase, older adults with cancer continued to use DHTs because they perceived them as useful for saving time and reducing the cost required for hospital visits. Participants also perceived technology to be easy to use and gained confidence as they continued to use it. Perceived usefulness and ease of use are well-established factors influencing individuals’ attitudes and intentions to use technology, as outlined in Technology Acceptance Models [30, 31] and studies on older adults with cancer [32, 33]. Our findings support prior literature and expand current understanding by identifying that these factors may facilitate not only technology acceptance but also continued use. These findings also suggest the importance of ensuring that DHTs are intuitive while underscoring that older adults may need ongoing support and encouragement to build confidence in using them.

Furthermore, the advantage of receiving timely care from healthcare providers through technology and the sense of increased self-control over their health status were identified as personal factors that facilitated the continued use of DHTs in older adults with cancer. Older adults with complex medical needs want to receive coordinated and patient-centered care and are likely to seek more information about their condition [22, 34]. Our findings suggest that the ability of DHTs to provide relatively rapid feedback on health-related concerns and customize care to fit individual requirements may have effectively met the unique care needs of older adults with cancer [10], potentially influencing their maintenance of DHT use after initial adoption. Perspectives shared during this study can inform healthcare providers and researchers about the direction for developing digital health tools that facilitate the continued use of DHTs among older adults with cancer. For instance, features providing real-time feedback and ensuring patient autonomy through information sharing can be seen as a possible way to enhance patient satisfaction and continued use of DHTs, compared to asynchronous and generic approaches [14, 32].

While most participants described positive perceptions of using DHTs for cancer management, some reported their lack of knowledge and skills in using technology independently, often requiring additional support from family members and contributing to limited use of DHTs. This supports the existing finding that low digital health literacy is the most significant barrier to the adoption and use of DHTs among older adults with cancer [35]. Older adults’ lack of knowledge and skills to use technology not only limits the effective implementation of DHT-based solutions but may also exacerbate existing disparities in cancer care, posing health equity risks [13, 26]. Therefore, healthcare providers should regularly assess patients’ level of digital literacy and provide additional support to increase their competencies regarding the use of DHTs in both the pre-and post-adoption phases [14]. It may also be helpful to promote the active use of DHTs among older adults with cancer by engaging older adults in the DHT design and implementation process, incorporating their perspectives, and increasing the fit between the technology and the patient’s lived experience [14].

Our study also found additional factors contributing to the limited use of DHTs in older adults with cancer. For some participants, their infrequent use of DHTs or desire to seek in-person care stemmed from a lack of direct interaction with healthcare providers and concerns about online communication. This is aligned with prior studies that reported older adults’ negative perceptions of telemedicine regarding limited personal connection with providers and reduced trust in the quality of care [36, 37]. It may suggest that technologies can be more appropriately considered as a supplement to, rather than a substitute for, traditional face-to-face care among this population, particularly in sensitive situations such as changes in symptoms or discussions around prognosis and end-of-life care [13]. To promote continued and active use of DHTs in older adults with cancer, healthcare providers should become aware of patients’ preferred ways of communication, provide flexibility in adjusting care delivery based on their needs, and ensure positive and high-quality patient experiences that meet or exceed those of in-person care [14, 38].

The findings of this study should be interpreted with consideration of several limitations. First, participants were recruited from a single cancer center and were mainly White (> 85%), which limits the transferability of our findings in other clinical contexts and cultures. Second, this study was based on a secondary data analysis of a larger qualitative study on general technological experiences among patients with cancer, and participants’ experiences were primarily limited to specific DHTs, such as virtual visits or patient portals. As a result, the findings may not fully capture the range and complexity of older adults’ unique perspectives and experiences with various DHTs. Lastly, the interviews were conducted in 2022. Given the rapid spread of digital health in geriatric cancer care and the increased familiarity with these technologies post-pandemic, future research is needed to explore additional factors affecting the initial adoption of DHTs among older adults.

## Conclusion

This study explored the perspectives of older cancer patients on using DHTs, focusing on factors affecting the adoption and use of DHTs. Despite digitalization in the healthcare system, technology accessibility remains a significant barrier to DHT adoption. It is required to ensure universal access to digital infrastructures and devices to facilitate the adoption of technology and reduce potential health inequity in cancer care. Most participants reported continued use of DHTs based on their perceptions of usefulness, ease of use, and satisfaction with specific features. However, additional efforts are needed to address the low digital literacy among older adults and their uncertainty regarding interaction and quality of care to facilitate more active and effective use of DHTs in cancer management.

## Data Availability

The data that support the findings of this study are available from the corresponding author upon reasonable request.
